# Focal brain trauma in the cryogenic lesion model in mice

**DOI:** 10.1186/2040-7378-4-6

**Published:** 2012-04-05

**Authors:** Furat Raslan, Christiane Albert-Weißenberger, Ralf-Ingo Ernestus, Christoph Kleinschnitz, Anna-Leena Sirén

**Affiliations:** 1 Department of Neurosurgery, University of Würzburg, 97080 Würzburg, Germany; 2 Department of Neurology, University of Würzburg, 97080 Würzburg, Germany

**Keywords:** Experimental brain trauma, Cryolesion, Secondary traumatic brain damage, Mice

## Abstract

The method to induce unilateral cryogenic lesions was first described in 1958 by Klatzo. We describe here an adaptation of this model that allows reliable measurement of lesion volume and vasogenic edema by 2, 3, 5-triphenyltetrazolium chloride-staining and Evans blue extravasation in mice. A copper or aluminium cylinder with a tip diameter of 2.5 mm is cooled with liquid nitrogen and placed on the exposed skull bone over the parietal cortex (coordinates from bregma: 1.5 mm posterior, 1.5 mm lateral). The tip diameter and the contact time between the tip and the parietal skull determine the extent of cryolesion. Due to an early damage of the blood brain barrier, the cryogenic cortical injury is characterized by vasogenic edema, marked brain swelling, and inflammation. The lesion grows during the first 24 hours, a process involving complex interactions between endothelial cells, immune cells, cerebral blood flow, and the intracranial pressure. These contribute substantially to the damage from the initial injury. The major advantage of the cryogenic lesion model is the circumscribed and highly reproducible lesion size and location.

## Introduction

Traumatic brain injury (TBI) and its sequel represent a major cause of disability and death worldwide [1]. TBI consists of the primary irreversible damage and a multitude of secondary cascades, resulting progressively in tissue degeneration and neurological impairment [2-4]. The outcome of severe cerebral lesion does not solely depend on the primary damage, but on the extent of secondary lesions.

Several models of experimental traumatic brain injury have been developed in an attempt to reproduce different aspects of the biomechanical impairments and neurological deficits observed in human head injury. The fluid percussion model produces brain injury by rapid injection of fluid into the closed cranial cavity [5,6]. The controlled cortical impact trauma is induced by using a pneumatic impactor to impact exposed brain with a measurable, controllable impact speed and cortical deformation [7]. In comparison to these two models of focal TBI, the unilateral cryogenic lesion model first described in 1958 by Klatzo [8] induces lesions that are well circumscribed and highly reproducible in size and location. Due to an early damage of the blood brain barrier (BBB), the cryogenic cortical injury leads to vasogenic edema, marked brain swelling, and inflammation. The histological and pathological features of cryogenic lesions share many of the hallmark characteristics of human TBI [9,10]. For example, upregulation of inflammatory cytokine expression is detected by real-time PCR as early as 24 h after cryogenic lesion [11]. The cryogenic lesion model can further be used to study long term neurodegenerative changes similar to late complications of human head injury such as global brain atrophy, cognitive impairment, and behavioural abnormalities [12,13]. Furthermore, cryogenic cortical injury has been shown to lead to late brain atrophy and impairment of spatial memory, two key readout parameters for late consequences of TBI [12]. Yet another advantage of the cryogenic model is its technical simplicity as craniotomy is not required.

## Material

### Experimental equipment

**Figure 1:**
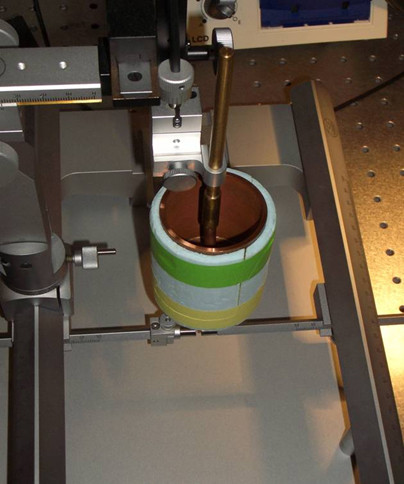
**Stereotaxic device**.

**Figure 2:**
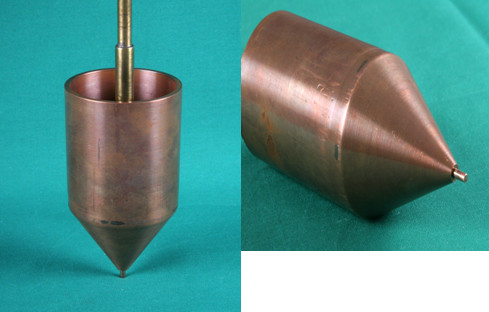
**Cone-shaped copper cylinder with tip diameter of 2.5 mm**.

1. Ketanest-S 25 mg/ml (S-ketamine hydrochloride) and Rompun 2% (xylazine)

2. Stereotaxic device (TSE Systems GmbH, Bad Homburg) depicted in Figure 1

3. Liquid nitrogen, Cotton-tipped applicators, 3-0 Silk sutures

4. Cone-shaped copper or aluminium cylinder with tip diameter of 2.5 mm and a body coated with polystyrene (Figure 2)

5. Heating device

### Setup of surgery and 2, 3, 5-triphenyltetrazolium chloride -staining

1. Scalpel (optional)

2. Scissors (e.g., delicate curved sharp/blunt iris scissors, Fine Science Tools Inc., Foster City, CA)

3. Forceps: One pair of splinter forceps (e.g., 90 mm curved splinter forceps, Aesculap AG, Tuttlingen, Germany)

4. Needle holder (e.g., Halsey Micro Needle Holder, Fine Science Tools)

5. Mouse brain matrix (e.g., mouse brain slicer matrix with 1 mm coronal slice intervals, Harvard Apparatus, Holliston, MA, USA)

6. Razor blade (e.g., SIH1 razor blades, Hartenstein Laborbedarf, Würzburg, Germany)

7. Phosphate-buffered saline (PBS)

8. 2, 3, 5-triphenyltetrazolium chloride (TTC) (e.g., Sigma-Aldrich, St. Louis, MO)

9. 2% Evans blue (Sigma)

9. ImageJ software (National Institutes of Health, USA) for planimetric calculation of lesion volumes

10. 4% paraformaldehyde, NaCl

## Methods

All animal experiments have to be conducted in accordance with the laws and regulations of the regulatory authorities for animal care, and they require an appropriate animal experimentation facility. Due to substantial strain and gender variations in mice, those crucial parameters are not interchangeable. In our laboratory, we use male, pathogen-free C57BL/6 mice (8-11 weeks) and genetically manipulated mice on a C57BL/6 background.

### Surgery

**Figure 3:**
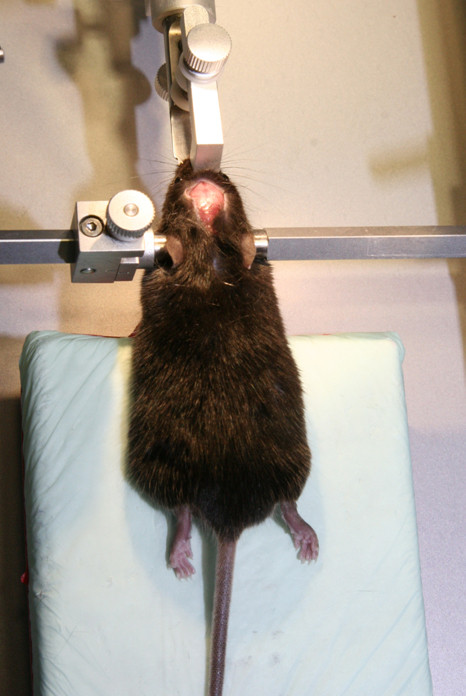
**3-point rigid cranial fixation in stereotaxic device**. A midline sagittal scalp incision is made to expose the skull bone.

**Figure 4:**
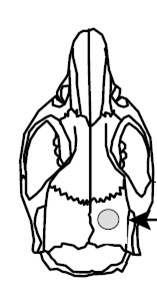
**Focal brain lesion coordinates from bregma: 1.5 mm posterior and 1.5 mm lateral**.

**Figure 5:**
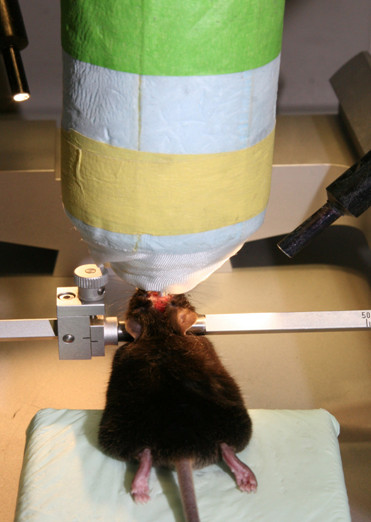
**Cone-shaped aluminium cylinder (tip diameter 2.5 mm) is filled with liquid nitrogen (-196°C) and placed on the right parietal skull coordinates as described in figure 4)**.

**Figure 6:**
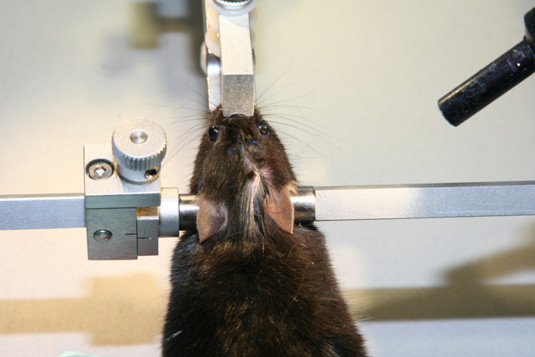
**Standard skin suture using, e.g., a needle holder and 3,0 Ethilon suture material**.

1. Anaesthesia with an intraperitoneal injection of Ketanest-S 25 mg/ml and Rompun 2% in a dose of 0.1 mg/g ketamine and 0.005 mg/g xylazine. The depth of surgical anaesthesia is verified before starting surgery.

2. 3-point rigid cranial fixation in stereotaxic device as depicted in Figure 3.

3. A midline sagittal scalp incision is made in the midline of the head (Figure 3).

4. The target area for lesion is on the right parietal skull, coordinates from bregma: 1.5 mm posterior, 1.5 mm lateral (Figure 4).

5. A cone-shaped copper or aluminium cylinder with a tip diameter of 1.5 or 2.5 mm will be filled with liquid nitrogen (-196°C) and placed stereotactically on the right parietal skull (coordinates as described) for definite time (60, 90, or 120 secs) (Figure 5). An extended contact time and more tip diameter range will increase the lesion volume. Sham-operated animals go through the same procedure without cooling the cylinder with liquid nitrogen.

6. The cone-shaped copper or aluminium cylinder is removed.

7. Wound closure by standard skin suture using a needle holder and, e.g., 3-0 Ethilon suture material (Figure 6).

The time required for surgery is usually 5-10 min.

### Outcome

Key readout parameters include the assessment of lesion size and the magnitude of blood brain barrier breakdown.

#### Determination of lesion size

**Figure 7:**
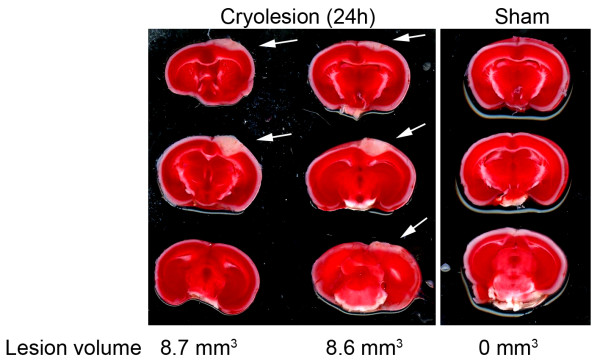
**Representative images of 2,3,5-triphenyltetrazolium chloride (TTC)-stained coronal brain sections from wild-type mice, two mice after cryogenic brain lesion (left) and one mouse after sham lesion (right) 24 h after cryogenic cortical injury or sham operation**. Planimetric measurements of lesion volumes are given for each case.

The cortical lesions increase over the first 24 h because the penumbral area surrounding the cryogenic cortical lesion is subsequently involved in the definite lesion area. 24 h after cryogenic cortical lesion induction, the brains are removed and sliced up into 2-mm-thick sections according to the coronal plane using a mouse brain slice matrix (Harvard Apparatus, Holliston, MA, USA). The slices are immersed in 2% TTC in PBS to visualize the lesion size (Figure 7). According to the calculated lesion areas with the known slice thickness lesion volumes can be measured planimetrically [11].

#### Determination of blood brain barrier permeability

Commonly occurring vasogenic edema formation, as sequel of breakdown of the BBB after traumatic brain injury, is one of the most important intracranial causes for secondary brain damage, leading to an increased intracranial pressure, causing brain shift, and at last brain herniation [14].

**Figure 8:**
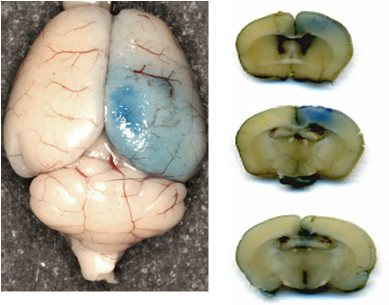
**Evan's Blue staining to assess the magnitude of the breakdown of the blood brain barrier after cryogenic injury**.

Further important read out criteria within this animal model is the assessment of the cerebral vasculature permeability after breakdown of the BBB to evaluate the traumatic vasogenic edema formation. This procedure can be easily and reliable investigated in the cryogenic cortical lesion model. 2% Evan's Blue tracer diluted in 0.9% NaCl is intravenously injected 2 h and 24 h after the induction of the cryogenic brain lesion. After 24 h the mice are killed with an overdose of the anesthetic and were transcardially perfused with 4% paraformaldehyde, the brains are removed and sliced up into 2-mm-thick sections according to the coronal plane using a mouse brain slice matrix. Planimetric measurements of the brain parenchyma stained with Evan's Blue (Figure 8) are used to calculate edema volumes [15]. According to the calculated lesion areas with the known slice thickness lesion volumes can be measured planimetrically [11,16].

The pearls and pitfalls of the cryogenic lesion models are listed below.

Advantages:

• highly standardized lesion size and location

• lesion volume on TTC is a reliable readout

• pronounced vasogenic edema and breakdown of the blood brain barrier that can easily be quantified by Evans blue extravasation

• technical simplicity, does not require craniotomy

• easily adaptable for mice

Disadvantages:

• not a mechanical trauma

• no counter coup, diffuse axonal injury or postcontusional bleeding

## Summary

Unilateral cryogenic brain lesion in mice with subsequent focal brain lesion and breakdown of the BBB is a valid model to investigate the histological and pathological characteristics of brain trauma and develop novel therapeutic strategies to reduce neuronal cell death and vasogenic brain edema [11]. Clinically relevant readouts that can be used for testing of new therapies in this model include lesion size, vasogenic edema, and inflammation.

## Competing interests

The authors declare that they have no competing interests.

## Authors' contributions

FR carried out the cryolesion experiments, performed data analysis, and drafted the manuscript. CK participated in the design and coordination of the study and supervised the experiments. RIE participated in the design of the study and edited the manuscript. CAW supported FR in performing the experiments and analyzing the data. ALS initiated, designed, and coordinated the study and finalized the manuscript. All authors read and approved the final manuscript.
